# In-depth analysis of *OTC* A208T case induced by *OTC* gene mutation and research on the prediction and simulation of the impact on protein function

**DOI:** 10.3389/fped.2024.1450859

**Published:** 2024-09-12

**Authors:** Qingyang Hu, Haiming Chen, Tianyi Liu, Xue Dong, Xuejiao Hu, Wenxin Yan, Zhong Li

**Affiliations:** ^1^ Department of Pharmacy, Dalian Women and Children’s Medical Group, Dalian, Liaoning, China; ^2^ College of Pharmacy, Dalian Medical University, Dalian, Liaoning, China; ^3^ Department of Emergency, Dalian Women and Children’s Medical Group, Dalian, Liaoning, China

**Keywords:** ornithine transcarbamylase deficiency, X-linked disorder, hyperammonemia, urea cycle disorder, molecular dynamics simulation

## Abstract

**Background:**

Ornithine transcarbamylase deficiency (OTCD), a rare hereditary disease caused by gene mutation of ornithine transcarbamylase (*OTC*), is the most prevalent type among urea cycle disorders. OTCD typically leads to mitochondrial enzyme dysfunction, preventing the synthesis of citrulline from carbamoyl phosphate and ornithine, and is characterized by a remarkable increase in blood ammonia. Specific symptoms may include neurological abnormalities, growth retardation, and other manifestations.

**Methods:**

We presented a case of a child diagnosed with OTCD (OMIM: 311250). By using whole-genome sequencing (WGS) for the pedigree and in-depth whole-exome sequencing (WES), we aimed to identify the disease-causing genes. Gene mutation prediction tools were employed to verify the pathogenicity, and the molecular dynamics simulation method was utilized to assess the impact of this mutation on the activity and structural stability of the *OTC* protein.

**Results:**

Whole-exome sequencing detected an *OTC* variant [NM_000531: c.622 (exon6) G > A, p.A208T]. Through comprehensive analysis with various gene mutation prediction tools and in line with the ACMG guidelines, this mutation site was firmly established as a pathogenic site. Moreover, the molecular dynamics simulation results clearly demonstrated that this mutation would significantly compromise the stability of the *OTC* protein structure.

**Conclusion:**

This study deepens our understanding of the clinical manifestations and characteristics of OTCD, especially the *OTC* A208T gene mutation site. Given the lack of specific clinical manifestations in OTCD patients, early and accurate diagnosis is crucial for effective treatment and prognosis improvement. To our knowledge, this is the first case of this mutation site reported in China.

## 1 Introduction

Ornithine transcarbamylase deficiency (OTCD) is a rare hereditary disorder resulting from mutations in the Ornithine transcarbamylase (*OTC*) gene, which exhibits an X-linked recessive or incomplete dominant inheritance pattern (1). The estimated prevalence of OTCD is between 1:14,000 and 80,000 (2), and it is the most common type among ornithine cycle disorders, accounting for 1/2–2/3 of ornithine cycle disorders (3). The *OTC* gene (NM_000531.5) is located at Xp11.4 on the chromosome (4), with a total length of 73 kb, consisting of 10 exons and 9 introns, and encoding 354 amino acids (5). This gene is mainly expressed in the liver and also expressed in intestinal mucosal cells.

When *OTC* gene mutations lead to reduced or absent *OTC* enzyme activity, the urea cycle is severely affected (6), resulting in the obstruction of citrulline synthesis and ornithine cycle. Then, it triggers ammonia degradation disorders and increases blood ammonia levels. The excessive accumulated ammonia has extremely strong toxicity to the central nervous system, which can interfere with the energy metabolism of brain cells, cause cytotoxic cerebral edema, neuronal apoptosis or atrophy, and affect the production of neurotransmitters in the brain, eventually leading to acute or chronic encephalopathy and neuropsychiatric damage. In addition, it will also lead to a large accumulation of carbamoyl phosphate and glutamine, and activate the pyrimidine metabolic pathway, increasing the generation and excretion of orotic acid. Clinically, this disease often presents as non-specific neurological damage and liver damage, so it is easily misdiagnosed as central nervous system infection or digestive system diseases.

Currently, the diagnosis of OTCD mainly depends on genetic testing, and the pathogenic mutations can occur at any position in the *OTC* gene. This paper reports a case of a newly occurred *OTC* missense mutation in China. The child had an acute onset and continuous increase of blood ammonia. The genetic test result showed that a missense mutation occurred in *OTC* A208T. The phenotypes of both the child's father and mother were normal, and the mother was a heterozygous mutation carrier at this site.

## 2 Materials and methods

### 2.1 Laboratory tests

#### 2.1.1 Blood tandem mass spectrometry (MS/MS) and urine gas chromatography-mass spectrometry (GC/MS)

Blood samples were collected using dry filter paper; blood amino acids and acylcarnitines were analyzed by blood tandem MS/MS. Urine was collected, and urine organic acids were analyzed by urine GC/MS.

Blood tandem MS/MS and urine gas chromatographic mass spectrometry were completed by Shenyang HH Medical Diagnostics Co., Ltd.

### 2.2 Detection of *OTC* gene mutations

#### 2.2.1 Whole-genome sequencing of the pedigree and deepened whole-exome sequencing of the pedigree

Whole-genome sequencing library construction: Extract DNA and construct the sequencing library through the random fragmentation method; Mutation screening: The sequencing library that has been constructed is sequenced on the machine, and the genomic coverage is not less than 99%; Genetic data analysis: Through the analysis and screening of the precision diagnosis cloud platform system for genetic diseases that integrates molecular biological annotation, biology, genetics and clinical characteristic analysis, combined with the pathogenic mutation database, normal human genome database, OMIM database and genetic data analysis algorithm, millions of genetic variations (non-coding region variations within the range of 159 bp upstream and downstream of the Han coding region and YTR region variations) are graded. The variation grading adopts the three-element grading system and the ACMG genetic variation grading system, and at the same time, annotate and analyze the possible structural variations.

#### 2.2.2 The gene mutation prediction tool predicts the mutation site

##### 2.2.2.1 Mutation taster

We utilized the online tool Mutation Taster (http://www.mutationtaster.org/) (7) to predict the potential impact of gene mutations. The prediction results are A: Possibly Disease causing automatic (possibly deleterious), D: possibly Disease causing (possibly deleterious), N: Polymorphism (possibly harmless), and P: Polymorphism automatic (possibly harmless). Among them, Disease causing automatic and Polymorphism automatic indicate that this variation has a record in the known database, and the evidence is apparent. We further investigated potential pathological variations based on the reports provided by this tool.

##### 2.2.2.2 polyPhen-2

We made use of the online genetic mutation prediction tool PolyPhen-2 (Polymorphism Phenotyping v2) (http://genetics.bwh.harvard.edu/pph2/) (8) to prognosticate if non-synonymous mutations would have a deleterious impact at the protein level. The prediction results are as follows: D: Probably damaging (score ≥0.909, highly likely to be harmful); P: Possibly damaging (0.447 ≤ score ≤0.909, potentially harmful); B: Benign (score ≤0.446, harmless). We carried out further investigations on potential pathological variations in accordance with the reports provided by this tool.

##### 2.2.2.3 SIFT

We used the Sorting Intolerant From Tolerant (SIFT) online tool (https://sift.bii.a-star.edu.sg/) (9) to carry out amino acid sequence conservation analysis and predict the potential impact of mutations on protein function. By inputting the protein sequence of *OTC* and the position and amino acid type of the putative mutation, the tool generates a score ranging from 0 to 1. Mutations with a score ≤0.05 are generally considered “deleterious” to protein function, while mutations with a score >0.05 are regarded as “benign”. These prediction results help us identify potential deleterious mutations for further investigation.

##### 2.2.2.4 FATHMM

We utilized the FATHMM tool (http://fathmm.biocompute.org.uk/) (10) to predict the potential impact of genetic sequence variations on protein function. The tool provides a numerical score based on its internal algorithm, with a low score (typically less than −1.5) indicating a potential negative impact of the mutation on protein function.

##### 2.2.2.5 PROVEAN

We employed the PROVEAN tool (http://provean.jcvi.org/index.php) (11) to evaluate the impact of predicted non-synonymous mutations on protein function. In accordance with the PROVEAN scoring system, variations with a score beneath the threshold of −2.5 are regarded as having a negative impact on protein function.

#### 2.2.3 Molecular dynamics simulation and structural stability prediction

We utilized the Alphafold v2.3.2 (12) developed by Deepmind to predict the protein structures of wild-type *OTC* and *OTC* mutation A208T.The monomeric_casp14 model in Alphafold v2.3.2 was used to predict the structure of the *OTC* protein, with all default parameters retained. The versions of the reference databases are as follows: the uniport and uniref90 databases were updated to March 1, 2023, the pdb_mmcif and pdb_seqres databases were updated to March 3, 2023, and the versions of other databases are default.

Molecular dynamics simulations based on GROMACS (2023.1 single-precision version) were performed (13). The experiments were conducted on a Dell T3660 workstation equipped with the Ubuntu 20.04.01 operating system. The heavy atoms and small molecules of the *OTC* protein were repaired using SPDBV 4.10 software (14). The topology structure of the *OTC* protein was constructed using the gmx pdb2gmx command, and the AMBER99SB-ILDN force field and the TIP3P water model were selected. Subsequently, the protein was placed in a 1 nm marginal radius and Na^+^ and Cl^−^ ions were added to a concentration of 0.15 M to achieve an electro-neutral state in a physiological saline solution environment. The system was energy-minimized and pre-equilibrated through restricted NVT and NPT for 100 ps to eliminate atomic conflicts. The steepest descent algorithm was used to perform 5,000 steps of energy minimization until the maximum force was below 1,000 kJ/mol/nm. The system reached equilibrium at 310 K and 1 bar pressure after 100 ps of restricted NVT and NPT pre-equilibration. After these initial steps, a total duration of 100 ns of MD simulation was performed, with a time step of 2 fs set by the Verlet buffer scheme and the trajectory data saved every 10 ps. After the simulation was completed, the “gmx trjconv” command was used to correct the trajectory of the protein movement and extract the spatial conformations at fixed time points. The “gmx rms” command is used to calculate the root mean square deviation (RMSD) of proteins. The “gmx RMSF” command is used to calculate the root mean square fluctuation (RMSF) of proteins. The free energy landscape map requires two conformation-related characteristic coordinates. In this experiment, RMSD and R_g_ (radius of gyration) are adopted as the horizontal and vertical coordinates for drawing the free energy landscape map.The covariance matrix was obtained through calculation using the R version 4.3.1 package “Bio3D” to measure the correlation between various variables (15).

## 3 Results

### 3.1 Case report

**Figure 1:**
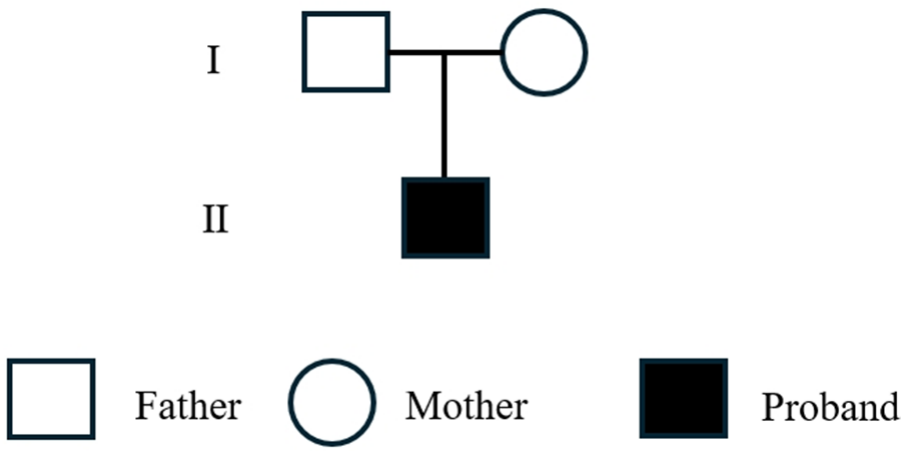
Pedigree of the proband.

The 14-year-old male patient manifested with an acute onset and a succinct disease course. After a preceding infection, there progressively occurred worsening multi-system symptoms, such as augmented drowsiness, restlessness, consciousness disturbances, and blurred vision. The physical examination outcomes demonstrated that the patient's conjunctiva exhibited mild swelling, and the bilateral Babinski sign was positive. Through lumbar puncture, it was ascertained that the intracranial pressure was elevated, yet the cerebrospinal fluid test results were normal, and the blood test signified coagulation dysfunction. Although the head magnetic resonance imaging failed to disclose any lesion, the patient's blood ammonia level anomalously rose and continuously increased. The patient customarily preferred a vegetarian diet and denied relevant family medical history as well as the situation of coma or vomiting after ingesting high-protein foods. Before the onset of the disease, the patient had the instance of consuming foods like chicken legs. The pedigree of the proband is shown in Figure 1.

### 3.2 Laboratory diagnosis

The routine test outcomes, encompassing the blood amino acid profile, acylcarnitine profile, and the detection results of organic acids on urine filter paper for this child, are presented in Table 1. The biochemical investigation finding indicates that the CK value of this child is 228 U/L, which exceeds the normal range, and the AMON value is 221 μmol/L, which is significantly augmented compared to the normal value. In accordance with the analysis report of the blood amino acid profile and acylcarnitine profile, the glutamic acid value of this child is 212 μmol/L, the methionine value is 72 μmol/L, and the glutamine value is 51.0 μmol/L, and all these three values are higher than the normal ranges. The examination results suggest that the blood ammonia of this child is prominently increased and shows a progressive increment. According to the analysis report of the detection of organic acids on urine filter paper, in this test result of this child, the value of uracil-2 is 24.27, and the data of acetylglycine-1 is 1.04, and both these two data points are higher than the normal values. Moreover, the value of orotic acid −3 is 0.00 (This value is the result after correcting hyperammonemia. The normal range is 0.00–2.50).

**Table 1:** Summary of laboratory test results for the pediatric patient.

Item	Result	Ref.
Arterial blood gas analysis		
pH	7.445	7.35–7.45
pCO_2_	35.7 mmHg	35.0–45.0
pO_2_	95.0 mmHg	83.0–108
Complete blood count		
CRP	<0.2 mg/L	0–10
PCT	<0.02 ng/ml	0–0.1
Biochemical investigation		
ALT	27 U/L	7–43
AST	23 U/L	12–37
Urea	4.6 mmol/L	2.7–7.0
Crea	50 μmol/L	37–93
<CK	228 U/L	0–200
AMON	221 μmol/L	9–30
Blood mass spectrometry analysis		
Glu	212 μmol/L	40–200
Met	72 μmol/L	8–35
Gln	51.0 μmol/L	2.5–30.0
Met/Phe	1.43	0.20–0.80
Determination of urinary organic acids		
Lactic acid-2	0.67	0.00–6.70
Acetylglycine-1	1.04	0.00–0.50
Uracil-2	24.27	0.00–19.65
Orotic acid-3	0.00	0.00–2.50

### 3.3 Detection of *OTC* gene mutations

#### 3.3.1 Whole-genome sequence of the pedigree and deepened whole-exome sequencing of the pedigree

The variant proband is a hemizygote, which is in line with the pathogenesis of X chromosome-linked inheritance (XL) disorders, and the impact of X chromosome inactivation ought to be taken into account. The chromosome locus where the mutation occurred in this child is chrX: 38262952; the position of the nucleotide alteration is c622 (exon6) G > A, signifying that the base G at position 622 of the coding region of the *OTC* gene mRNA has mutated to A and is situated in the sixth exon; the position of the amino acid alteration is: p.A208T (p.Ala208 Thr) (NM_000531), that is, at the position of the 208th amino acid of the protein encoded by the *OTC* gene, alanine is mutated to threonine. Additionally, in accordance with the ACMG guidelines (2019), the variation is likely to be pathogenic. Based on the above genetic test results, it implies that the harmfulness of the *OTC* gene variation in this case is associated with the patient's phenotype.

#### 3.3.2 Mutation site prediction using genetic mutation prediction tools

##### 3.3.2.1 Mutation taster

c.622 (exon6) G > A. The base G at position 622 of the Exon 6 of the gene mRNA has mutated to A, with a mutation rate of 0.99997826, and the predicted outcome is deleterious.

##### 3.3.2.2 polyPhen-2

p.A208T (p. Ala 208 Thr). The 208th amino acid of this gene is mutated from alanine to threonine, which might be disruptive, with a score of 0.999.

##### 3.3.2.3 SIFT

p.A208T, with a score of 0.05, and is predicted to be a deleterious gene.

##### 3.3.2.4 FATHMM

The prediction result for p.A208T is deleterious.

##### 3.3.2.5 PROVEAN

The prediction result of p.A208T is −2.84000 and it is deleterious. The results of mutation site prediction by genetic mutation prediction tools are shown in Table 2.

**Table 2:** The prediction results of the gene mutation prediction tool.

Predict tool	Score	Predict result
Mutation Taster	0.9999	Disease causing
polyPhen-2	0.999	Probably damaging
SIFT	0.05	damaging
FATHMM	−5.37	damaging
PROVEAN	−2.84	Deleterious

### 3.4 Molecular dynamics simulation

**Figure 2:**
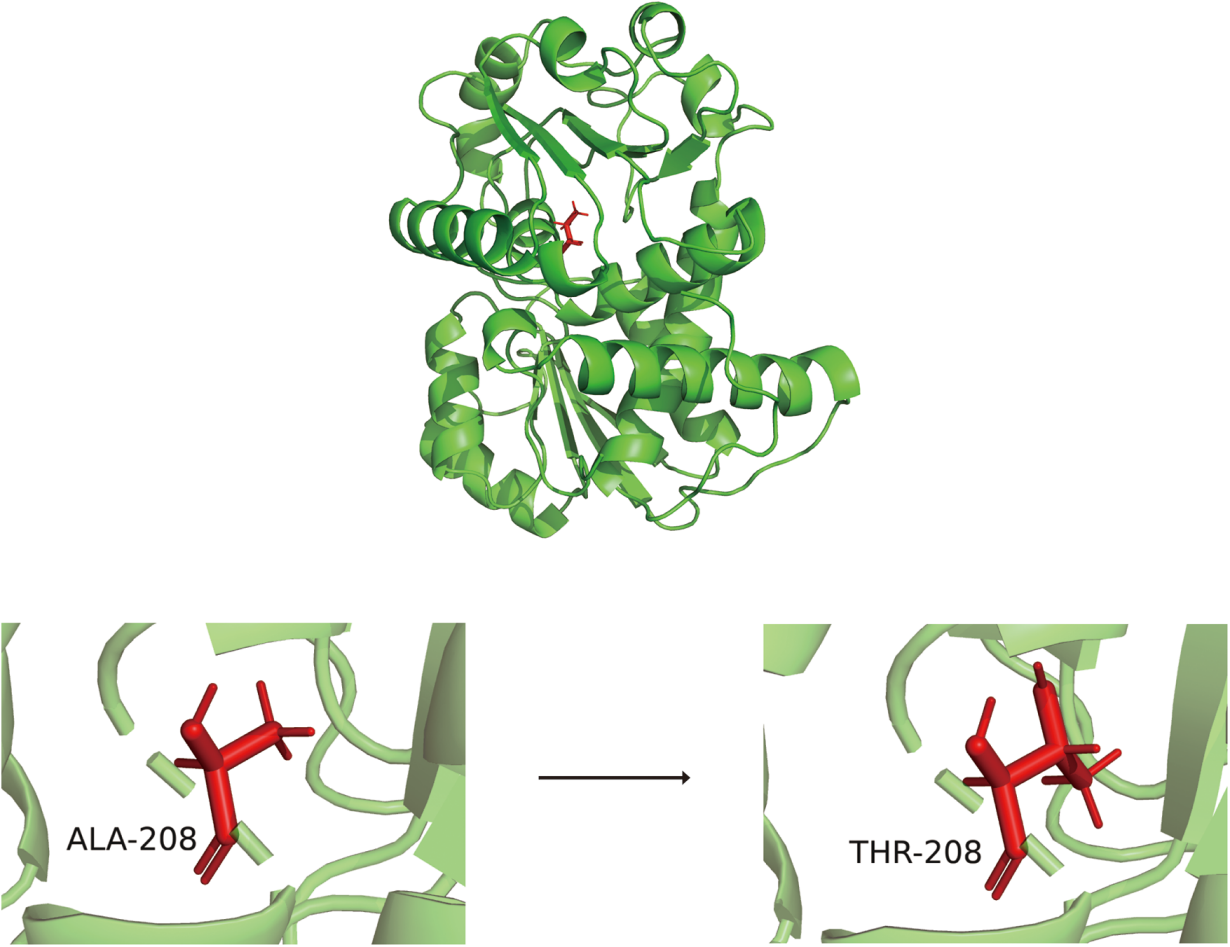
The three-dimensional structure diagrams of wild-type OTC and OTC mutation A208T protein.

Alphafold 2.3.2 was employed to predict the 3D structures of the wild-type *OTC* and the *OTC* mutation A208T proteins, and subsequently, the alterations in RMSD, RMSF, FEL, and PCA during the molecular dynamics simulation procedure were utilized to analyze the changing situation of the structural stability of the *OTC* mutation A208T protein. The structural alterations of the wild-type *OTC* and the *OTC* mutation A208T proteins are presented as Figure 2.

#### 3.4.1 RMSD

**Figure 3:**
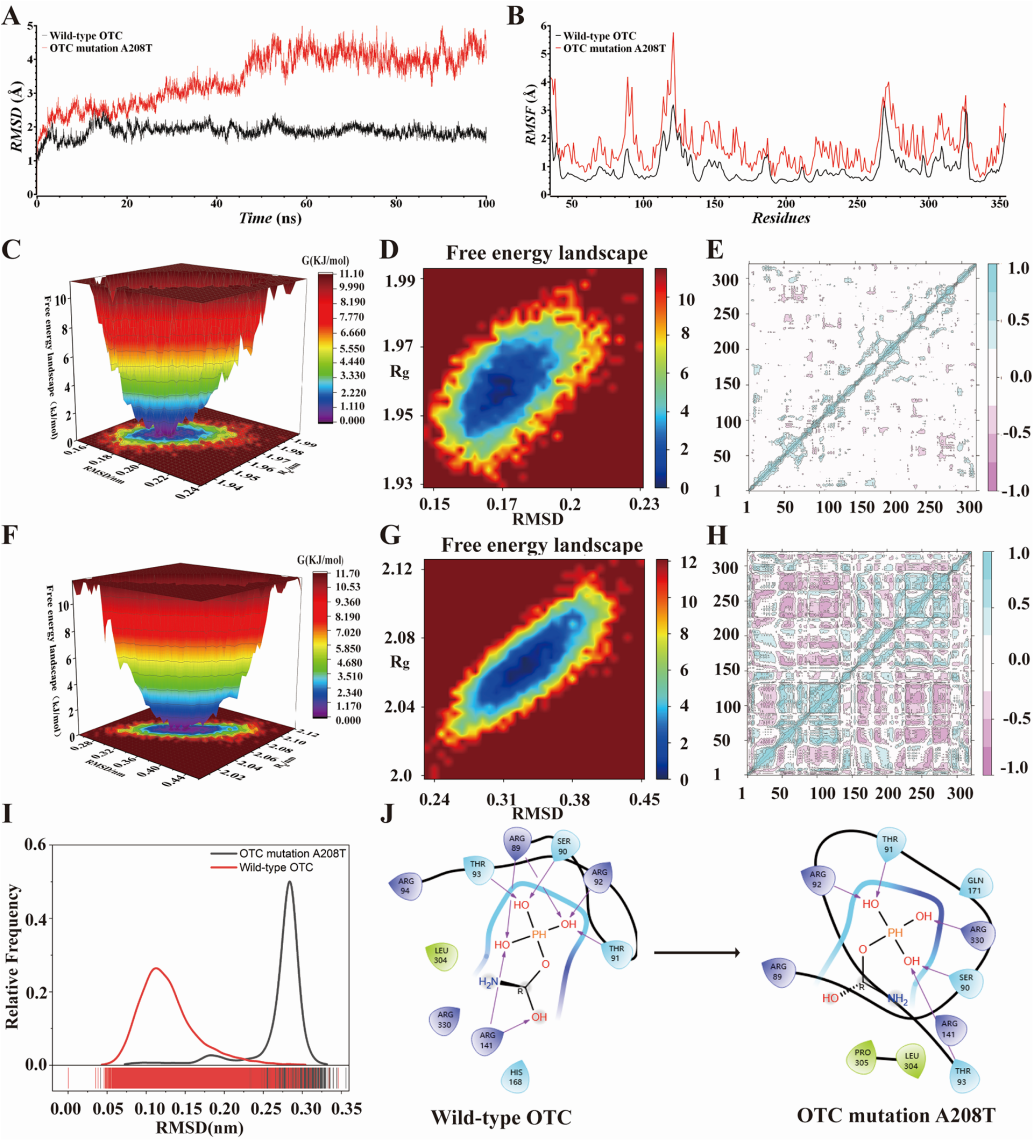
**(A,B)** RMSD and RMSF graphs of wild-type OTC and OTC mutation A208T during 100 ns MD simulation. **(C–G)** 3D and 2D FEL of the OTC and the OTC mutation A208T during 50–100 ns MD simulation. **(E,H)** PCA graphs of wild-type OTC and the OTC mutation A208T. **(I)** The normal distribution graph drawn by the RMSD of wild-type OTC and OTC mutation A208T during the 100 ns MD simulation. **(J)** The 2D combination mode of flexible molecular docking between carbamoyl phosphate and wild-type OTC and OTC mutation A208T.

In order to more effectively assess the stability of the system, we computed the root mean square deviation (RMSD) of the wild-type *OTC* and the *OTC* mutation A208T. In contrast to the stable RMSD value of the wild-type *OTC*, the all-atom main chain RMSD value of *OTC* A208T exhibits an upward tendency, suggesting the presence of an instable effect. As depicted in Figure 3A, after 20 ns of simulation, the RMSD value of the normal *OTC* remains within a narrow fluctuation range, less than 1 Å; however, the RMSD value of the *OTC* mutation A208T fluctuates continuously and fails to reach an equilibrium within the 100 ns simulation time. These data not only signify that the A208T mutation has a profound impact on the structural stability of *OTC*, but also that it might have an influence on the biological function of *OTC*. In the later stage of the simulation process, the RMSD value of *OTC* A208T progressively increases, and within the last 10 ns of the simulation time, the maximum deviation value attains 1.7 Å. And the average RMSD value of *OTC* A208T (3.5 Å) is significantly greater than the average RMSD value of the wild-type *OTC* (1.9 Å), indicating that the mutation A208T significantly alters the topological structure of the protein.

#### 3.4.2 RMSF

Meanwhile, we also computed the root mean square fluctuation (RMSF) of the carbon backbone of the wild-type *OTC* and the *OTC* mutation A208T in order to assess the stability of the system. In comparison with the RMSF value of the wild-type *OTC*, the all-atom main chain RMSF value of *OTC* A208T keeps on rising, indicating the presence of an unstable effect. As presented in Figure 3B, within the residue ranges of 88–95, 116–122, 141–149, 163–171, and 270–279, the RMSF of *OTC* A208T fluctuates more prominently when compared to the RMSF value of the wild-type *OTC*, all exceeding 1 Å.

#### 3.4.3 FEL

When appraising the free energy change of the molecular conformation of the wild-type *OTC* and its *OTC* mutation A208T, we utilized the free energy landscape map to portray their energy states. By analyzing the energy troughs in these graphs, it can be deduced whether the binding of the receptor and the ligand attains the lowest energy equilibrium state. Figures 3C–D illustrate that the wild-type *OTC* exhibits a deep and single energy valley in the free energy landscape map, suggesting that its molecular structure is relatively stable. In contrast, Figures 3F–G the energy valley in the free energy landscape map of the *OTC* mutation A208T is shallower, indicating that this mutation might lead to the instability of the protein structure and is more prone to being affected by external factors, thereby triggering structural deformation or degradation.

#### 3.4.4 PCA

We carried out PCA on the wild-type *OTC* and the *OTC* mutation A208T, and drew the PCA graph to observe the distribution of the data points. In the PCA graph of the wild-type *OTC*, the data points are predominantly concentrated in the blue area, indicating that the majority of the variations have a coherent pattern and trend within this area. Comparatively, there are relatively fewer points in the red area, suggesting the existence of some minor or aberrant changes. In the PCA graph of the *OTC* mutation A208T, the distribution of the data points is more complex. Although the blue area remains the main concentration area, the number of data points in the red area has significantly increased. Figures 3E,H illustrate that in comparison to the wild-type *OTC*, this mutation has introduced more structural or functional diversities. These red areas might reflect the impact of the mutation on the *OTC* protein, causing some originally inconspicuous variations or characteristics to become more prominent.

#### 3.4.5 MD simulation of the binding of wild-type *OTC* and *OTC* mutation A208t to carbamoyl phosphate

The ornithine cycle is an absolutely crucial approach for ammonia degradation within the organism. *OTC* predominantly resides in mitochondria and is capable of potently catalyzing the reaction between ornithine and carbamoyl phosphate to generate citrulline, which will then be translocated to the cytoplasm to further participate in the ornithine cycle. It should be emphasized that the ornithine cycle is not a single simplistic reaction, but rather consists of multiple interrelated steps, involving the synergistic effects of various enzymes and substances. Once the *OTC* gene undergoes mutation, there is a high probability of a significantly reduced or even complete absence of the activity of the *OTC* enzyme. As a result, both the synthesis of citrulline and the progression of the ornithine cycle will be severely impeded, thereby giving rise to obstacles in ammonia degradation and ultimately leading to abnormally elevated blood ammonia levels. Excessive elevation of blood ammonia may trigger serious disorders such as hepatic encephalopathy, exerting significant harm to brain functions, and may also cause varying degrees of damage to multiple systems and organs of the body, like the nervous system and digestive system, giving rise to a series of complex pathophysiological alterations.

MD simulations were respectively carried out for carbamoyl phosphate, the wild-type *OTC* and its combination with the *OTC* mutation A208T. As can be seen from the normal distribution graph drawn by RMSD in Figure 3I, the RMSD value of the *OTC* mutation A208T is relatively higher than that of the wild-type *OTC*. We also conducted molecular docking for carbamoyl phosphate, the wild-type *OTC* and its combination with the *OTC* mutation A208T. The 2D binding pattern diagram is presented as Figure 3J. When carbamoyl phosphate binds to the wild-type *OTC*, the three -OH of the phosphate group form hydrogen bonds with THR93, SER90, THR91, ARG89, ARG92and ARG141 respectively, and the C-OH forms a hydrogen bond with ARG141; when carbamoyl phosphate binds to the mutant *OTC* A208, the three -OH on the phosphate group form hydrogen bonds with ARG330, SER90, THR93, THR91 and ARG92 respectively, and there is no hydrogen bond formation on the C-OH.

### 3.5 Treatment and follow-up

Upon the child's admission to the hospital, multiple treatment measures, including hypothermic brain protection, infusion of arginine, a low-protein diet, catheterization, and facilitation of defecation to reduce blood ammonia, were promptly administered. Five days after the treatment, the child's body temperature stabilized, consciousness was regained, the mental state became clear, and the plasma ammonia dropped to the normal level. Eight days after the treatment, the child was discharged upon reaching the clinical cure standard. Communication was established with the patient via telephone. The parents of the child indicated that the current physical condition of the child is favorable, and there has been no recurrence of disease-related symptoms. Subsequently, regular communication with the patient will be maintained, and continuous attention will be given to the health status of the patient. In case of any abnormality, corresponding treatments will be promptly implemented.

The diagnosis of OTCD depends on the determination of *OTC* enzyme activity in liver biopsy or peripheral blood gene mutation detection. Hepatic biopsy and enzyme analysis are also of paramount significance and irreplaceability for the diagnosis of OTCD. It can profoundly analyze the specific conditions of enzymes in liver tissue, thereby clearly and precisely determining whether the patient has OTCD. It not only facilitates the early detection of the disease but also provides detailed and reliable information for subsequent treatment decisions, and plays a vital role in monitoring the progression of the disease and evaluating the treatment effect.

In the treatment of the acute phase of OTCD, it is extremely crucial to control hyperammonemia timely and effectively. The treatment means mainly include dietary control, drug therapy, and liver transplantation. In terms of diet, it is advisable to ingest low-protein and high-calorie nutritious foods. The purpose of dietary control is to reduce the intake of protein to lower ammonia production. In the aspect of drug therapy, commonly used drugs such as sodium benzoate and sodium phenylacetate are used to promote the excretion of ammonia in the body. In addition, it is also possible to maintain the acidic environment of the intestinal tract by taking oral lactulose or using vinegar enema, while supplementing arginine and citrulline to promote the urea cycle. For patients with severe hyperammonemia, hemofiltration is the preferred treatment option. Compared with hemodialysis, hemofiltration possesses distinct advantages in pathophysiological mechanisms. Hemodialysis mainly removes small molecular substances through diffusion, and its efficacy in removing medium and large molecular substances is limited. For patients with hyperammonemia, not only is there small molecular ammonia but also some medium and large molecular metabolites, which might aggravate the disease or influence the treatment effect. Hemofiltration can slowly and continuously remove small and medium molecular solutes and water through convection, and at the same time, it also has a certain ability to remove medium and large molecular substances. In particular, continuous veno-venous hemofiltration (CVVH) and continuous veno-venous hemodiafiltration (CVVHDF). CVVH can stably excrete excessive ammonia and avoid drastic fluctuations in the internal environment, thus making it suitable for patients with underlying diseases or those who are physically frail. CVVHDF combines the advantages of hemodialysis and filtration, which can not only accurately remove small molecular substances such as ammonia but also remove medium and large molecular substances, comprehensively dealing with the metabolic disorders caused by hyperammonemia.

For the patient population in the current study, given the severity of their hyperammonemia and possible complex conditions, CVVH and CVVHDF have outstanding adaptability (16). There exists a substantial accumulation of metabolic wastes, including ammonia in the patient's body, and there is an extremely high demand for the safety and effectiveness of treatment. Hemofiltration can more accurately target the pathological characteristics of hyperammonemia. These blood purification methods hold great significance in the treatment of hyperammonemia. Adopting these hemofiltration methods can effectively reduce the ammonia content in the patient's blood and alleviate the neurological damage, coma and other symptoms caused by hyperammonemia. During the treatment process, vital signs and blood indicators need to be closely monitored, and the plan should be adjusted in time according to the specific situation to ensure safety and effectiveness. In the follow-up stage, attention should be continuously paid to the rehabilitation situation, and regular examinations should be conducted to prevent the recurrence of hyperammonemia and provide a guarantee for the long-term health of patients. In some extremely severe cases, liver transplantation may be the only effective means to address abnormal liver function (17). For children with severe episodes of neonatal OTCD and those with late-onset OTCD patients with ineffective conservative treatment, living donor liver transplantation is a feasible treatment option, which can significantly improve the prognosis of the patients. Although liver transplantation can fundamentally solve the problem, it involves many challenges, such as donor matching, surgical risks, and long-term postoperative management, etc., so careful consideration is required. At the same time, dietary control and drug therapy should be precisely adjusted according to the specific situation of the patient to achieve the best therapeutic effect, thereby maximizing the survival quality and health status of the patient.

## 4 Discussion

OTCD is a rare hereditary disorder and a type of urea cycle abnormality (18). When the *OTC* gene undergoes mutation, it leads to abnormal structure and function of the encoded *OTC*, thereby interfering with the normal progression of the urea cycle. This results in metabolic wastes such as ammonia within the body not being effectively transformed and excreted, and accumulating in the body, subsequently triggering a series of severe symptoms like hyperammonemia and neurological damage, ultimately leading to the occurrence of OTCD.

In this study, we presented a patient with OTCD, presenting characteristics including confusion of consciousness, headache, drowsiness, encephalitis, abnormal coenzyme activity, restlessness, and avoidance of protein foods. Laboratory examination findings demonstrated that the patient had severe hyperammonemia, an increase in CK level in the blood, a significant rise in AMON level, and an increase in the levels of Glu, Gln, Met, and Met/Phe. The levels of acetylglycine-1 and uracil-2 in urine also increased. The value of orotic acid −3 is 0.00. This value is the result after correcting hyperammonemia and is within the normal range. Hence, we conducted whole-genome high-throughput sequencing for the family and deepened whole-exome sequencing for the family to determine the genetic cause of this congenital metabolic abnormality. Exome sequencing and genetic data analysis indicated that the phenotypic manifestations of the patient might be attributed to the mutation of the *OTC* gene. The genetic test results showed that the variant was a hemizygote in the proband, which was in line with the pathogenesis of X chromosome-linked genetic (XL) diseases; the phenotypes and genotypes of the proband and its family members were in accordance with cosegregation, taking into account the influence of X chromosome inactivation. The mutation site we studied is similar to the mutation report result of this site that has been reported in the past (19–23).

Traditionally, we directly conduct sequencing of the *OTC* gene or utilize related gene mutation prediction tools to predict the pathogenicity of mutation sites. In this study, we also adopted computational chemistry methods and *OTC* deeply explored the structure and stability of wild-type *OTC* and *OTC* mutation A208T protein through molecular dynamics simulations. Three-dimensional modeling of *OTC* and *OTC* A208T mutant was carried out using Alphafold software, and through multiple methods such as RMSD, RMSF, FEL, and PCA for simulation, it was predicted that the stability of *OTC* mutation A208T is poorer than that of wild-type *OTC* and cannot remain stable during the 100-ns simulation process. The stability of proteins will impact the biological function of *OTC*, thereby continuously influencing the urea cycle.

In the urea cycle, *OTC* catalyzes the reaction between ornithine and carbamoyl phosphate to generate citrulline, which is a crucial step in the urea cycle (24). The generated citrulline will continue to participate in subsequent reactions, and ultimately ammonia will be excreted out of the body, thereby realizing the metabolism and detoxification of ammonia. However, mutations in the *OTC* gene will cause a reduction or loss of *OTC* enzyme activity, and then lead to ornithine cycle disorder diseases. We combined wild-type *OTC* and *OTC* mutation A208T with carbamoyl phosphate respectively, and the obtained RMSD graph indicates that the RMSD value of *OTC* mutation A208T is relatively high, which suggests that the structural fluctuation of *OTC* mutation A208T during the simulation process is relatively large, and the binding with carbamoyl phosphate may be less stable. And the 2D binding pattern diagram of carbamoyl phosphate with wild-type *OTC* and its combination with *OTC* mutation A208T shows that more hydrogen bonds are formed when carbamoyl phosphate binds to wild-type *OTC*, which may imply that the interaction between wild-type *OTC* and carbamoyl phosphate is stronger and the binding is tighter. In addition, in wild-type *OTC*, the C-OH forms a hydrogen bond with ARG141, while in *OTC* mutation A208T, the C-OH does not form a hydrogen bond, which may also affect the binding strength and stability between the two. These differences indicate that there are structural and property changes when *OTC* mutation A208T binds to carbamoyl phosphate compared to wild-type *OTC*, which may have an impact on their functions and biological activities. Although molecular dynamics simulation cannot fully reflect the real situation of the mutation occurrence, it can clearly show the dynamic trend of proteins and provides important verification support for our research.

## 5 Conclusion

The existing research findings comprehensively depict the disease traits of OTCD resulting from the *OTC* gene. The mutation data at the *OTC* [c.622 (exon6) G > A, p. A208T] site of this patient possesses significant reference significance for families with this kind of disease in aspects such as genetic diagnosis and consultation, as well as clinical meticulous management, which is conducive to facilitating the realization of early diagnosis of OTCD and preventing the occurrence of misdiagnosis.

## Data Availability

The original contributions presented in the study are included in the article/Supplementary Material, further inquiries can be directed to the corresponding author.
